# Functional Characterization of the Basal Amygdala-Dorsal BNST Pathway during Contextual Fear Conditioning

**DOI:** 10.1523/ENEURO.0163-20.2020

**Published:** 2020-07-13

**Authors:** Jennifer Sasaki Russell, Stéphanie Trouche, Leon G. Reijmers

**Affiliations:** 1 Department of Neuroscience, School of Medicine, Tufts University, Boston, MA 02111; 2 Program in Neuroscience, Graduate School of Biomedical Sciences, Tufts University, Boston, MA 02111; 3 IGF, University of Montpellier, CNRS, INSERM, Montpellier, France 34094

**Keywords:** contextual fear, fear memory, basal amygdala, bed nucleus of the stria terminalis, neural circuits, encoding, fear engram

## Abstract

Both the basal amygdala (BA) and the bed nucleus of the stria terminalis (BNST) can participate in contextual fear, but it is unclear whether contextual fear engrams involve a direct interaction between these two brain regions. To determine whether dorsal BNST (dBNST)-projecting neurons in the BA participate in contextual fear engrams, we combined the TetTag mouse with a retrograde tracer to label dBNST-projecting cells in the BA. We identified a population of neurons located in the anterior subdivision of the BA (aBA) that was activated during fear conditioning and reactivated during retrieval but that did not project to the dBNST. In contrast, dBNST-projecting neurons located in the posterior BA (pBA) were activated during contextual fear conditioning but were not reactivated during retrieval. Similarly, we found neurons in the oval BNST subdivision (ovBNST) that were activated during contextual fear conditioning without being reactivated during retrieval. However, the anterodorsal BNST (adBNST) subdivision was not activated during either contextual fear conditioning or retrieval, underscoring the divergent functionality of these two dBNST subdivisions. Finally, we found that the ovBNST receives a monosynaptic projection from neurons located in the BA. Our results indicate that aBA neurons that do not project to the dBNST participate in contextual fear engrams. In contrast, dBNST-projecting neurons in the BA do not appear to participate in contextual fear engrams, but might instead contain a BA → ovBNST pathway that is active during the initial encoding of contextual fear memories.

## Visual Abstract

**Figure:**
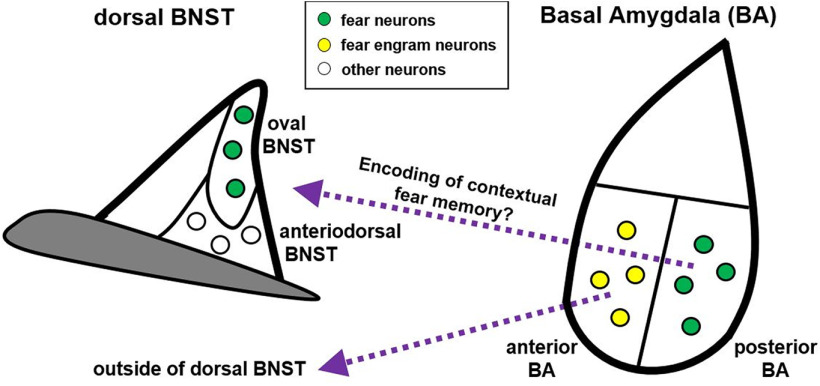

## Significance Statement

Both the basal amygdala (BA) and the dorsal bed nucleus of the stria terminalis (dBNST) can participate in contextual fear, but it is unclear whether this reflects a direct interaction between these two brain regions. BA neurons that do not project to the dBNST were found to be active during both the encoding and retrieval of a contextual fear memory, indicating their participation in a contextual fear engram. In contrast, BA neurons that do project to the dBNST were found to be active during the encoding, but not the retrieval of a contextual fear memory. These findings suggest a direct interaction between the BA and dBNST during the initial encoding, but not the subsequent storage of contextual fear memories.

## Introduction

A context previously paired with an aversive experience can induce a sustained and anxiety-like fear that is associated with similar brain activation patterns across vertebrate species (Alvarez et al., 2008). Identifying the specific neural substrates of contextual fear in rodents can therefore lead to a better understanding of the mechanisms that might underlie anxiety and fear disorders in humans (Davis et al., 2010). Previous studies have implicated both the basal amygdala (BA) and the bed nucleus of the stria terminalis (BNST) in contextual fear (Goosens and Maren, 2001; Sullivan et al., 2004; Resstel et al., 2008; Duvarci et al., 2009; Onishi and Xavier, 2010; Poulos et al., 2010; Zimmerman and Maren, 2011). Although a direct projection from the BA to the BNST has been reported (Dong et al., 2001a), it is unclear whether the BA and BNST directly interact with each other during the encoding and/or expression of contextual fear.

Both the BA and BNST are composed of subnuclei with distinct functional heterogeneity and connectivity. The BA is divided into the anterior BA (aBA) and the posterior BA (pBA), which have been shown to contribute differently to the support of a memory dependent on emotional valence (Kim et al., 2016). The dorsal BNST (dBNST) includes the oval, anterodorsal, and mediodorsal BNST (ovBNST, adBNST, and mdBNST), with the ovBNST and adBNST exhibiting opposing functions in anxiety (Kim et al., 2013). While studies indicate both structures contribute to contextual fear, the high level of connectivity between the amygdala and BNST, and between each structure’s own subdivisions, make it challenging to isolate long-range circuits and determine their function in the contextual fear engram.

In an effort to characterize the role of BA-BNST circuitry in contextual fear memory, we used a transgenic mouse called the TetTag mouse to fluorescently tag neurons in the BA and the dBNST that were activated during contextual fear conditioning (i.e., fear neurons), and combined this with immunolabeling of an immediate early gene (IEG) to detect neuronal activity on re-exposure to the context. This allowed us to look for BA and dBNST neurons that were active during both fear conditioning and retrieval sessions, which would indicate their participation in a contextual fear engram (i.e., engram neurons). We combined this TetTag approach with circuit-tracing to identify the possible participation of BA → dBNST projecting neurons in contextual fear engrams.

Our findings identify a population of cells in the aBA that participate in the contextual fear engram but that do not project to the dBNST. A separate population of dBNST-projecting cells in the pBA were found to be activated during contextual fear conditioning without being reactivated during retrieval. Interestingly, our functional and anatomic data indicate the possibility that these pBA → dBNST neurons might activate neurons located in the ovBNST subdivision during contextual fear conditioning.

## Materials and Methods

All animal procedures were performed in accordance with the National Institutes of Health Guide for the Care and Use of Laboratory Animals and were approved by Tufts University Animal Care and Use Committee. Adult female and male TetTag mice were used, with each experimental group consisting of approximately equal numbers of females and males. As no sex differences were observed, the data from females and males were combined. Mice were housed with water and regular doxycycline chow (40-mg doxycycline/kg) provided *ad libitum* and were individually housed beginning immediately after surgery, or one week before fear conditioning in non-surgical cases. Doxycycline chow was replaced with standard chow (no doxycycline) for all mice (fear conditioned group and home cage group) 4 d before fear conditioning. High doxycycline chow (1-g doxycycline/kg) was provided for all mice overnight following the conclusion of fear conditioning, then replaced with regular doxycycline chow for the remainder of the experiment.

### Stereotactic surgeries

TetTag mice were stereotactically injected with either 45 nl (for ovBNST) or 150 nl (for dBNST) of 0.05% cholera toxin subunit B (CTB) Alexa Fluor 647 conjugate (Invitrogen, C-34778) dissolved in sterile PBS. Bilateral injections were performed to target the dBNST (AP +0.65 mm, ML ±1.1 mm, DV −4.25) using a 0.5-μl Neuros Syringe (Hamilton, 7000.5) fitted with a 32-gauge blunt-point needle. The CTB solution was injected at a rate of 100 nl/min. The needle was slowly removed 10 min after the completion of injection. Mice were given a 10-d postsurgery recovery period before starting behavioral experiments.

C57BL/6 mice were stereotactically injected with 300 nl of AAV2-CMV-GFP (Virovek, 0.625E + 13 vg/ml). Bilateral injections were performed to target the posterior BA (AP −1.35, ML ±3.45, DV −5.2) using a 5-μl syringe (Hamilton, 75RN) fitted with a 33-gauge blunt-point needle. The AAV was injected at a rate of 100 nl/min. The needle was slowly removed 10 min after the completion of injection. Mice were killed two weeks after injection to allow sufficient time for virus expression.

### Behavior

On day 1 of the behavioral experiment, mice were subjected to contextual fear conditioning, which consisted of three training sessions each separated by 3 h. At the start of each fear conditioning session, the mouse was transferred to a plexi-glass box with a grid floor (Coulbourn Instruments, H10-11RTC, 120 wide × 100 deep × 120 high) contained within an isolation chamber. Foot shocks (2 s each, 0.70 mA) were delivered at 198, 278, 358, and 438 s, with a total session time of 500 s. Mice were returned to their home cage in between each session. On day 4, mice were subjected to a 500-s retrieval test. Mice were placed in the context used for fear conditioning but did not receive foot shocks during the testing session. The sessions were recorded with an above digital camera and freezing behavior was quantified using Actimetrics FreezeFrame software. The bout length of freezing was set to 1 s, and the threshold for freezing was determined by an experimenter blinded to group. Mice in the home cage group remained in their cage throughout the duration of the experiment.

### Tissue preparation and immunohistochemistry

Mice were anesthetized and transcardially perfused with ice cold 4% paraformaldehyde 80 min after the start of the retrieval session. Home cage mice, which did not undergo a retrieval session, were perfused on the same day, staggered between perfusions of fear conditioned mice throughout the day. Brains were dissected and postfixed overnight in 4% paraformaldehyde, then sunk in 30% sucrose for 3 d. Brains were sliced into 20-μm coronal sections on a cryostat. Free-floating tissue sections were rinsed three times for 15 min in PBS with 0.25% Triton X-100 (PBS-T), then transferred to a blocking solution of PBS-T with 10% normal goat serum for 1 h at room temperature. Sections were incubated in a primary antibody solution of rabbit anti-Zif268 (Santa Cruz, polyclonal; 1:3000), or rabbit anti-SynapsinI (ThermoScientific; polyclonal; 1:1000) combined with mouse anti-PSD95 (Pierce Antibodies; monoclonal; 1:500). Primary antibodies were diluted in the blocking solution, incubated at 4°C for 72 h, and rinsed three times for 15 min in PBS-T. Secondary antibodies (Jackson ImmunoResearch; goat anti-rabbit 549, 1:1500, goat anti-mouse 647, 1:500) were diluted in the blocking solution and applied to the sections for 2 h at room temperature. Sections were mounted on slides and cover-slipped using DAPI mounting media to label cell nuclei and stored at 4°C.

### Microscopy

Images for the BNST TetTag experiment were acquired on an epifluorescent TissueFAXS Whole Slide Scanning System using a 20× air objective.

All other images were acquired on a Nikon A1R confocal laser scanning microscope using a 20× air, 40× oil, or 60× oil objective. Image stacks were acquired at 2-μm step sizes for a total of 8–10 *Z* sections per image field. The maximum intensity projection image was used for subsequent analysis.

### Image analysis

For the BNST TetTag experiment, image analysis was performed manually by identifying and counting GFP+, Zif+, or GFP+Zif+ cells, which were normalized to the number of DAPI cells [estimated by region of interest (ROI) area] for each subdivision.

For the CTB TetTag experiment, image analysis was performed using ImageJ software. Quantification of GFP+ and Zif+ cells were done by thresholding the image and filtering for particle size. Masks of GFP+, Zif+, and colocalizing GFP+Zif+ cells were superimposed on the corresponding CTB image and GFP+Zif+CTB– and GFP+Zif+CTB+ cells were manually identified and counted for each section. Left and right BA were analyzed for each section, with three to six sections per mouse. Final percentages of cell types are averages per mouse. Chance level of colocalization between GFP and Zif was calculated as [(total number of GFP+)/(total number of base cells)] × [(total number of Zif+)/(total number of base cells)] × 100%. Base cells were either DAPI, CTB+, or CTB– cells. Comparison with chance level was used to identify engram neurons that have significant reactivation that cannot be explained by random overlap of GFP and Zif (Reijmers et al., 2007; Tayler et al., 2013; Trouche et al., 2013).

Synaptic colocalization was analyzed using Imaris 3D imaging software. Colocalizing synaptic markers were identified by creating “spots” based on SynapsinI puncta and filtering for spots that contained above-threshold intensity pixels for PSD-95 puncta. A 3D mask of GFP+ axons was used to select for overlapping spots.

### Statistics

Data were analyzed in GraphPad Prism 6. Datasets were tested for normality with the Shapiro–Wilk test. Paired or unpaired two-tailed *t* tests were used for normally distributed variables to evaluate significance with *p* < 0.05 as the level of statistical significance. Data that were not normally distributed were analyzed with non-parametric tests: the Mann–Whitney test for unpaired mean comparisons and the Wilcoxon matched signed-rank test for paired mean comparisons. Data in graphs are presented as mean ± SEM. Normality, type of test, group means with SEM, and *p* value for data comparisons are presented in Table 1.

**Table 1:** Statistical table

Figure	Brainregion	Item	Population	Group	Normality	Mean (±SEM)	Type of test	*p* value	Significance(*p* < 0.05)
2	aBA	a	GFP+ (%DAPI)	HC	Yes	0.864 (0.158)	Unpaired *t* test	0.0069	Yes
				FC	Yes	1.895 (0.277)			
		b	Zif+ (%DAPI)	HC	Yes	8.635 (1.214)	Unpaired *t* test	0.2388	No
				FC	Yes	10.75 (1.211)			
		c	GFP+Zif+ (%DAPI) vs chance	HC Chance	Yes	0.078 (0.020)	Paired *t* test	0.7966	No
				HC	Yes	0.070 (0.037)			
				FC Chance	Yes	0.192 (0.026)	Paired *t* test	0.0163	Yes
				FC	Yes	0.479 (0.092)			
		d	GFP+Zif+ minus chance level	HC	Yes	–0.009 (0.032)	Unpaired *t* test	0.0133	Yes
				FC	Yes	0.287 (0.095)			
		e	GFP+CTB- cells (% CTB- cells)	HC	Yes	0.803 (0.146)	Unpaired *t* test	0.0043	Yes
				FC	Yes	1.840 (0.260)			
		f	Zif+CTB- (% CTB- cells)	HC	Yes	6.516 (0.997)	Unpaired *t* test	0.0484	Yes
				FC	Yes	9.688 (1.072)			
		g	GFP+Zif+CTB- (% CTB- cells) vs chance	HC Chance	No	0.068 (0.017)	Paired Wilcoxon	0.3828	No
				HC	No	0.042 (0.026)			
				FC Chance	Yes	0.177 (0.039)	Paired *t* test	0.0301	Yes
				FC	Yes	0.431 (0.073)			
		h	GFP+Zif+CTB- minus chance level	HC	Yes	–0.013 (0.027)	Unpaired *t* test	0.0082	Yes
				FC	Yes	0.261 (0.081)			
		i	GFP+CTB+ cells (% CTB+ cells)	HC	Yes	0.966 (0.250)	Unpaired *t* test	0.145	No
				FC	Yes	1.750 (0.425)			
		j	Zif+CTB+ (% CTB+ cells)	HC	Yes	17.32 (2.527)	Unpaired *t* test	0.651	No
				FC	Yes	15.82 (2.081)			
		k	GFP+Zif+CTB+ (% CTB+ cells) vs chance	HC Chance	Yes	0.171 (0.056)	Paired Wilcoxon	0.6406	No
				HC	No	0.186 (0.136)			
				FC Chance	Yes	0.256 (0.074)	Paired *t* test	0.1383	No
				FC	Yes	0.478 (0.142)			
		l	GFP+Zif+CTB+ minus chance level	HC	Yes	0.015 (0.102)	Unpaired *t* test	0.2482	No
				FC	Yes	0.223 (0.135)			
3	pBA	m	GFP+ (% DAPI cells)	HC	Yes	1.629 (0.133)	Unpaired *t* test	0.0005	Yes
				FC	Yes	2.884 (0.243)			
		n	Zif+ (% DAPI cells)	HC	Yes	6.399 (0.924)	Unpaired *t* test	0.8923	No
				FC	Yes	6.574 (0.872)			
		o	GFP+Zif+ (%DAPI) vs chance	HC Chance	Yes	0.101 (0.015)	Paired *t* test	0.4835	No
				HC	Yes	0.126 (0.045)			
				FC Chance	Yes	0.190 (0.030)	Paired *t* test	0.2324	No
				FC	Yes	0.234 (0.036)			
		p	GFP+Zif+ minus chance level	HC	Yes	0.025 (0.034)	Unpaired *t* test	0.6978	No
				FC	Yes	0.044 (0.034)			
		q	GFP+CTB- cells (% CTB- cells)	HC	Yes	1.547 (0.1740)	Unpaired *t* test	0.0036	Yes
				FC	Yes	2.729 (0.2838)			
		r	Zif+CTB- (% CTB- cells)	HC	Yes	4.748 (0.653)	Unpaired *t* test	0.4069	No
				FC	Yes	5.529 (0.638)			
		s	GFP+Zif+CTB- (% CTB- cells) vs chance	HC Chance	Yes	0.073 (0.011)	Paired *t* test	0.5843	No
				HC	Yes	0.062 (0.020)			
				FC Chance	Yes	0.150 (0.021)	Paired *t* test	0.5166	No
				FC	Yes	0.175 (0.040)			
		t	GFP+Zif+CTB- minus chance level	HC	Yes	–0.011 (0.020)	Unpaired *t* test	0.4118	No
				FC	Yes	0.025(0.037)			
		u	GFP+CTB+ cells (% CTB+ cells)	HC	Yes	2.163 (0.430)	Unpaired *t* test	0.0249	Yes
				FC	Yes	4.24 (0.685)			
		v	Zif+CTB+ (% CTB+ cells)	HC	Yes	13.47 (2.282)	Unpaired *t* test	0.4735	No
				FC	Yes	11.37 (1.784)			
		w	GFP+Zif+CTB+ (% CTB+ cells) vs chance	HC Chance	No	0.269 (0.078)	Paired Wilcoxon	0.8438	No
				HC	No	0.545 (0.324)			
				FC Chance	Yes	0.447 (0.080)	Paired Wilcoxon	0.8203	No
				FC	No	0.658 (0.301)			
		x	GFP+Zif+CTB+ minus chance level	HC	No	0.277 (0.255)	Mann- Whitney	0.4807	No
				FC	No	0.211 (0.283)				
4	ovBNST	y	GFP+ (%DAPI)	HC	Yes	5.015 (0.858)	Unpaired *t* test	0.0106	Yes
				FC	Yes	11.07 (1.808)			
		Z	Zif+ (%DAPI)	HC	Yes	11.04 (2.986)	Mann- Whitney	0.9015	No
				FC	No	15.79 (7.200)			
		aa	GFP+Zif+ (%DAPI) vs chance	HC Chance	Yes	0.630 (0.250)	Paired Wilcoxon	0.0156	Yes
				HC	No	0.399 (0.225)			
				FC Chance	No	2.38 (1.28)	Paired Wilcoxon	0.0938	No
				FC	No	1.48 (0.750)			
		bb	GFP+Zif+ minus chance level	HC	Yes	–0.232 (0.061)	Mann-Whitney	0.62	No
				FC	No	–0.896 (0.561)			
	adBNST	cc	GFP+ (%DAPI)	HC	Yes	7.656 (1.883)	Unpaired *t* test	0.542	No
				FC	Yes	9.517 (2.290)			
		dd	Zif+ (%DAPI)	HC	No	9.385 (4.003)	Mann-Whitney	0.714	No
				FC	Yes	14.99 (6.305)			
		ee	GFP+Zif+ (%DAPI) vs chance	HC Chance	No	0.896 (0.501)	Paired Wilcoxon	0.5781	No
				HC	No	1.069 (0.709)			
				FC Chance	No	1.948 (1.267)	Paired Wilcoxon	0.5625	No
				FC	Yes	0.939 (0.397)			
		ff	GFP+Zif+ minus chance level	HC	No	0.173 (0.295)	Mann-Whitney	>0.9999	No
				FC	No	–1.009 (1.052)			

Statistical information for Figures 2-4.

## Results

**Figure 1.**
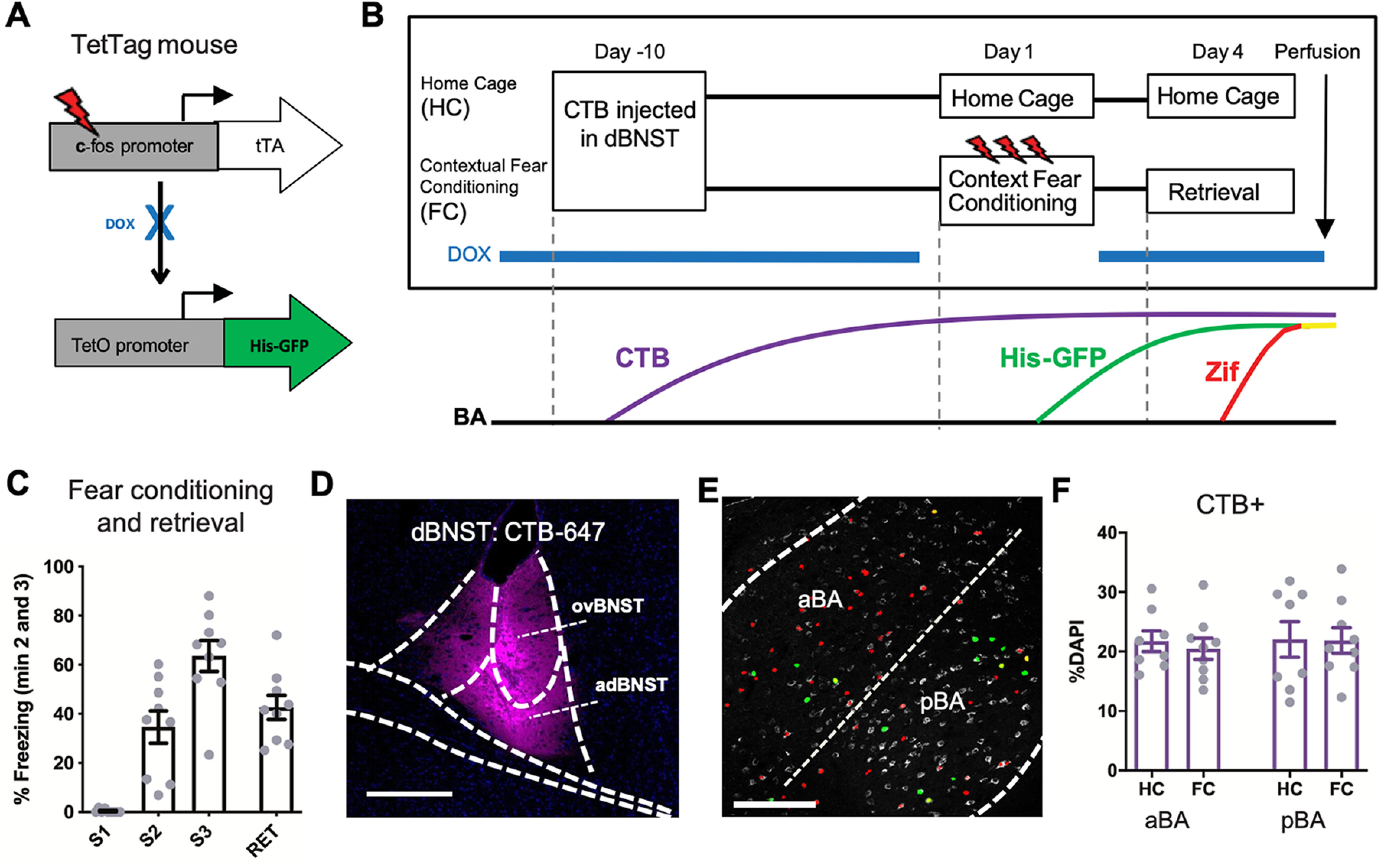
The TetTag mouse and retrograde tracing are combined to investigate BA-dBNST circuitry function in contextual fear memory. ***A***, Schematic of the double transgenic TetTag mouse line. Tetracycline transcription activator (tTA) is expressed under control of the c-fos promoter. In the absence of doxycycline (DOX), tTA binds to the tet operator (TetO) in the second transgene and drives expression of histone2B-GFP (His-GFP). His-GFP is long lasting and localized to the nucleus of cells activated during the off-DOX period. ***B***, Experimental paradigm for the CTB-injected FC group (*n* = 9) versus HC group (*n* = 8). Lower panel shows the corresponding expression timeline of the CTB, His-GFP, Zif. ***C***, The FC group showed typical levels of freezing during fear conditioning trials (S1, S2, S3) and retrieval session (RET). Freezing percentages are an average of the second and third minutes of each session. ***D***, CTB-647 expression near the injection site in the dBNST. Scale bar = 200 μm. ***E***, CTB-positive cells (CTB+, grayscale) in the BA with Zif mask (red), GFP+ mask (green), and colocalizing GFP+Zif+ cells (yellow). Scale bar = 100 μm. ***F***, Percentage of CTB+ cells among DAPI cells in the aBA and pBA were similar between FC and HC groups. Graphs show means ± SEM.

The BA is an important site for the generation, storage, and retrieval of fear memories and is richly connected with other brain regions that support the fear engram. To determine whether a subset of fear engram neurons in the BA project to the dBNST, we used the TetTag transgenic mouse, a *c-fos*-based reporter mouse (Fig. 1*A*), in combination with the retrograde tracer CTB to identify both fear neurons and dBNST-projecting neurons in the BA simultaneously. The TetTag mouse allowed us to tag neurons that were activated during the period of fear conditioning (i.e., fear neurons), while an IEG marker, *egr-1/zif268* (Zif), was used to identify fear neurons that were reactivated during retrieval of the fear memory (i.e., neurons likely part of the fear engram). Combined, the GFP and Zif markers provide two temporally defined activation profiles for the same set of cells within the brain ROI, allowing us to distinguish cells that were activated during fear conditioning alone, retrieval alone, or during both. One group of mice was submitted to a contextual fear conditioning paradigm (FC group, *n* = 9), while the other group remained in their home cage as controls (HC group, *n* = 8). The FC group was assessed for contextual fear memory 3 d later in a retrieval session and perfused shortly after to allow for sufficient Zif expression (Fig. 1*B*). All mice in the FC group showed normal levels of freezing during fear conditioning trials and the retrieval session (Fig. 1*C*). CTB injections were confirmed to target the adBNST and the ovBNST within the dBNST (Fig. 1*D*). During our preliminary analysis, we observed a notable difference in CTB intensity and pattern between the anterior (aBA) and posterior (pBA) subdivisions of the BA (Fig. 1*E*). This led us to quantify the data separately for these two subdivisions, as they might contain parallel BA → dBNST pathways with different properties. The average percentage of CTB–labeled cells was similar across groups for both BA subdivisions (Fig. 1*F*).

**Figure 2.**
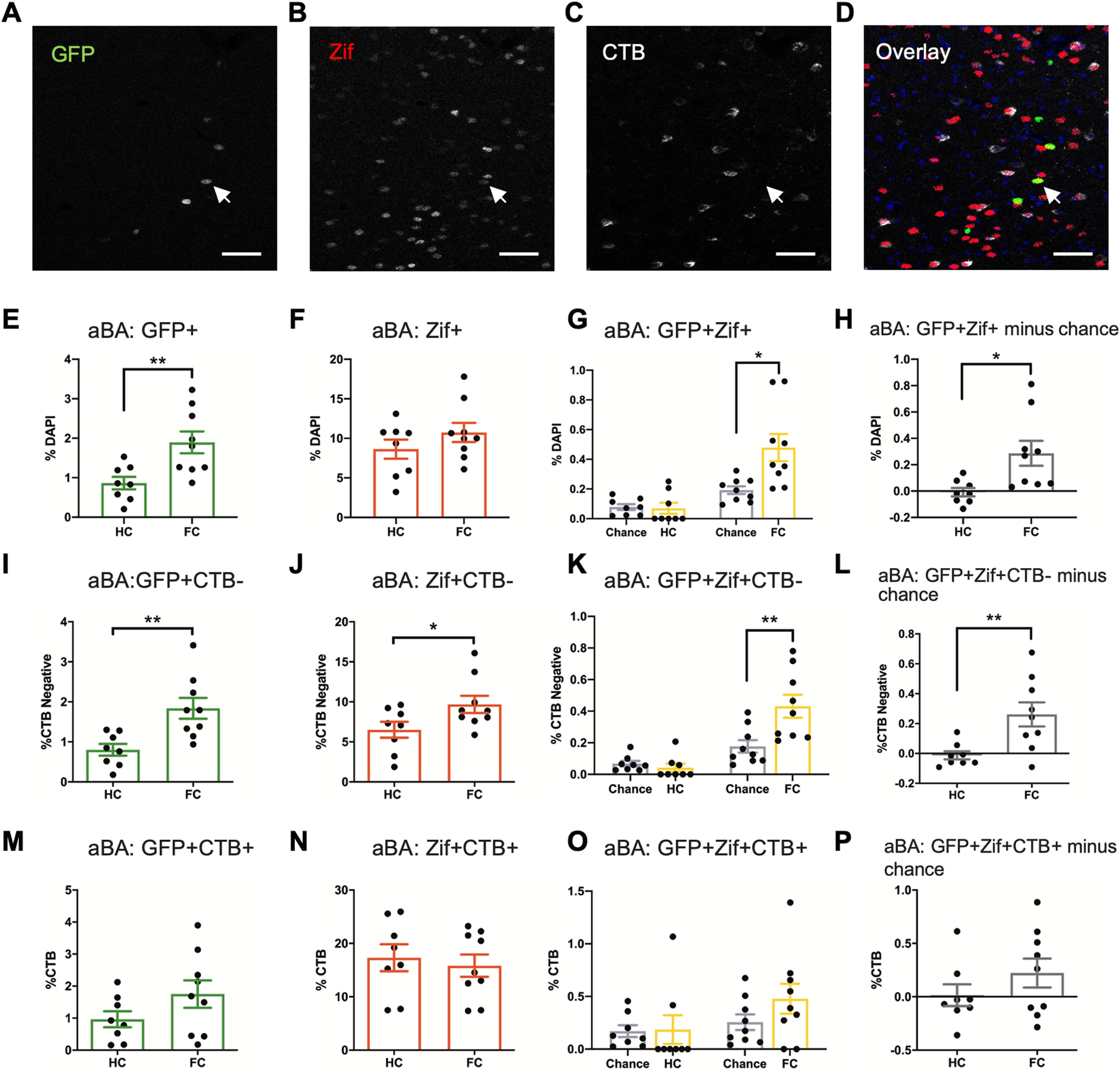
Contextual fear engram neurons in the aBA do not project to the dBNST. ***A–D***, Representative images of (***A***) GFP, (***B***) Zif, (***C***) CTB, and (***D***) overlay from a section of aBA with DAPI (blue). White arrow indicates a GFP+Zif+CTB– cell. Overlay displays an example of masks from thresholded images of GFP and Zif that were used for quantification and to determine colocalization. Scale bar = 50 μm. ***E***, Contextual fear conditioning increased the percentage of GFP+ neurons among DAPI cells in the aBA (*p* = 0.0069). ***F***, There was no difference between FC and HC in total percentage of Zif+ neurons in the aBA (*p* = 0.24) due to fear retrieval. ***G***, Fear conditioning and retrieval increased the percentage of GFP+Zif+ reactivated fear neurons compared with chance level in the aBA (*p* = 0.016). ***H***, The number of GFP+Zif+ cells minus chance level was significantly higher in the FC group compared with HC in the aBA (*p* = 0.013). ***I***, Contextual fear conditioning increased the percentage of GFP+CTB– neurons among CTB negative (CTB–) cells in the aBA (*p* = 0.0043). ***J***, Fear retrieval increased the percentage of Zif+CTB– neurons among CTB– cells in the aBA (*p* = 0.048). ***K***, Fear conditioning and retrieval increased the percentage of GFP+Zif+CTB– reactivated fear neurons among CTB– cells in the aBA compared with chance level (HC: *p* = 0.38; FC: *p* = 0.030). ***L***, The number of GFP+Zif+CTB– cells minus chance level was significantly higher in the FC group, indicating reactivation of fear neurons during retrieval (*p* = 0.0082). ***M–P***, Fear conditioning and retrieval did not significantly change any levels of activated cells in the CTB+ population in the aBA; ***M***, GFP+CTB+ (*p* = 0.15); ***N***, Zif+CTB+ (*p* = 0.65); ***O***, GFP+Zif+CTB+ versus chance (HC: *p* = 0.64; FC: *p* = 0.14); ***P***, GFP+Zif+CTB+ cells minus chance level (*p* = 0.25). Graphs show mean ± SEM. *p < 0.05, **p < 0.01.

We first analyzed total activation levels of the aBA, then further divided the analysis into subgroups dependent on CTB labeling to determine projection type (Fig. 2*A–D*). There was a significant increase in total GFP+ cells, but not Zif+ cells, in the aBA of the FC group (GFP+ *p* = 0.0069^a^, Zif+ *p* = 0.24^b^; Fig. 2*E*,*F*). The FC group also had a higher level of GFP+Zif+ colocalizing cells than chance level, while the HC group did not (FC vs chance, *p* = 0.016; HC vs chance, *p* = 0.80^c^; Fig. 2*G*), and GFP+Zif+ minus chance level was higher in the FC group than HC group (*p* = 0.013^d^; Fig. 2*H*). Next, we analyzed activation levels of non-dBNST-projecting fear neurons (CTB–) in the aBA. We found an increase in both GFP+ and Zif+ neurons in the FC group vs HC group (GFP+CTB– *p* = 0.0043^e^; Zif+CTB– *p* = 0.048^f^; Fig. 2*I*,*J*). Colocalization analysis showed that the FC group had significantly more fear neurons that were reactivated during the retrieval session (GFP+Zif+CTB–) than chance level, while the HC group did not (FC vs chance, *p* = 0.030^g^; HC vs chance, *p* = 0.38; Fig. 2*K*). There were significantly more reactivated fear neurons minus chance level in the FC group than in the HC group (*p* = 0.0082 h; Fig. 2*L*). These GFP+Zif+CTB– cells in the aBA likely participate in a circuit between the BA and one or more downstream structures that supports the storage of the fear memory (Reijmers et al., 2007). Conversely, the dBNST-projecting cells in the aBA (CTB+) showed similar activation levels between the FC and HC groups across all profiles (GFP+CTB+ *p* = 0.15^i^; Zif+CTB+ *p* = 0.65^j^; GFP+Zif+ CTB+ HC vs chance *p* = 0.64, FC vs chance *p* = 0.14^k^; GFP+Zif+CTB+ minus chance level, HC vs FC *p* = 0.25^l^; Fig. 2*M–P*).

**Figure 3.**
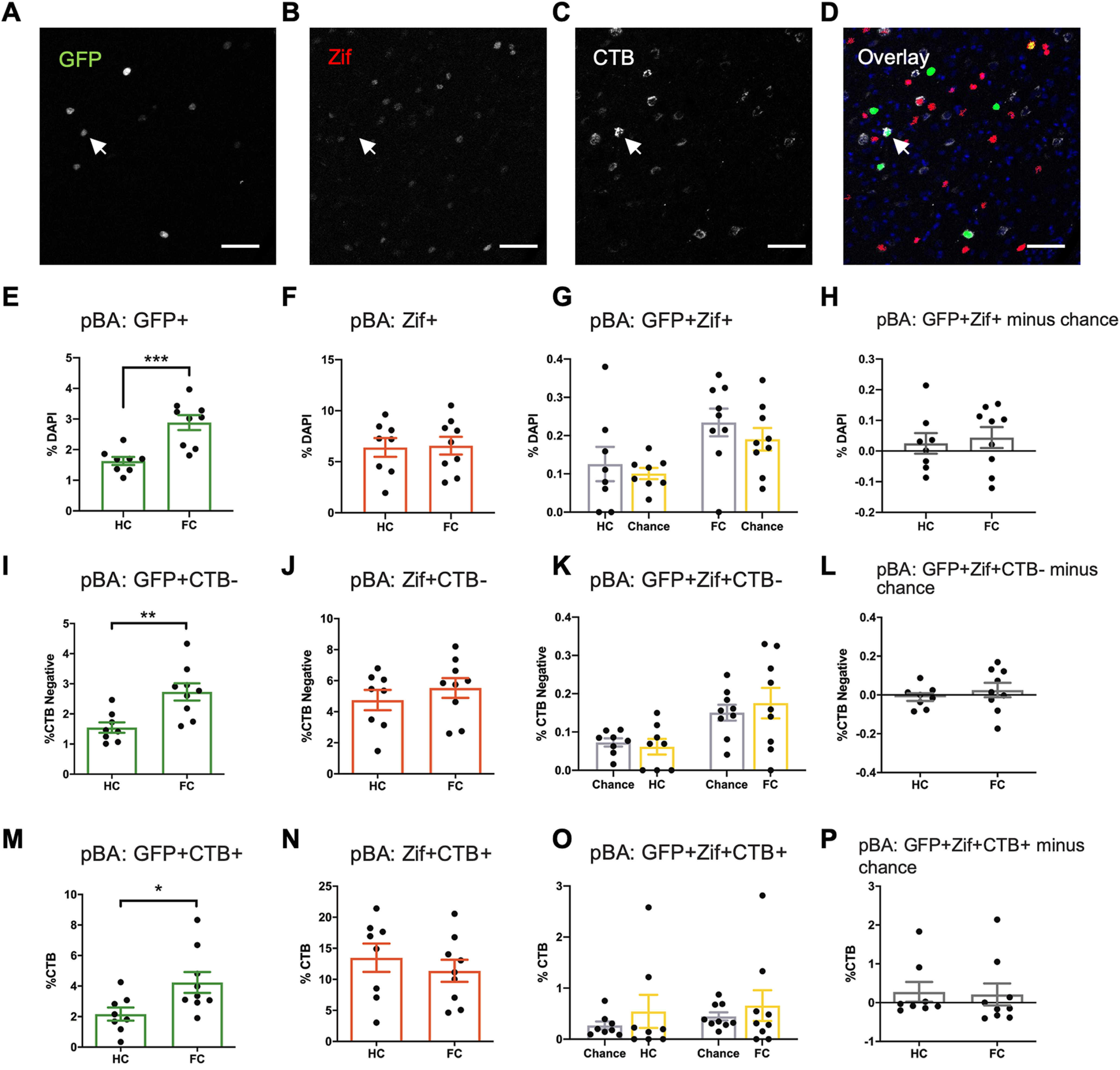
The pBA-dBNST circuit is active during contextual fear conditioning, but is not incorporated into the fear engram. ***A–D***, Representative images of GFP, Zif, CTB, and overlay from a section of pBA with DAPI (blue). White arrow indicates a GFP+Zif-CTB+ cell. Scale bar = 50 μm. ***E***, Contextual fear conditioning increased the percentage of GFP+ neurons among DAPI cells in the pBA (*p* = 0.0005). ***F***, There was no difference between FC and HC in total percentage of Zif+ neurons in the pBA (*p* = 0.89) due to fear retrieval. ***G***, Fear conditioning and retrieval did not increase the percentage of GFP+Zif+ reactivated fear neurons compared with chance level in the pBA (*p* = 0.23). ***H***, The number of GFP+Zif+ cells minus chance level was not significantly different in the FC group compared with HC in the pBA (*p* = 0.70). ***I–L***, Fear conditioning increased levels of GFP+ in the CTB– population in the pBA, but Zif+ and GFP+Zif+ did not differ between the groups; ***E***, GFP+CTB– (*p* = 0.0036); ***F***, Zif+CTB– (*p* = 0.41); ***G***, GFP+Zif+CTB– versus chance (HC: *p* = 0.58; FC: *p* = 0.52); ***H***, GFP+Zif+CTB– cells minus chance (*p* = 0.42). ***M–P***, Fear conditioning increased levels of GFP+ in the CTB+ population in the pBA, but Zif+ and GFP+Zif+ did not differ between the groups; ***M***, GFP+CTB+ (*p* = 0.025); ***N***, Zif+CTB+ (*p* = 0.47); ***O***, GFP+Zif+CTB+ versus chance (HC: *p* = 0.84; FC: *p* = 0.82); ***P***, GFP+Zif+CTB+ cells minus chance level (*p* = 0.48). Graphs show mean ± SEM. *p < 0.05, **p < 0.01, ***p < 0.001.

We next analyzed the activation patterns of the pBA (Fig. 3*A–D*). In contrast to the aBA, the pBA contained only increased numbers of GFP+ cells in the FC group compared with HC (GFP+ *p* = 0.0005^m^; Zif+ *p* = 0.89^n^; GFP+Zif+ HC vs chance *p* = 0.48, FC vs chance *p* = 0.23°; GFP+Zif+ minus chance level, HC vs FC *p* = 0.70^p^; Fig. 3*E–H*). Similarly, the non-dBNST-projecting neurons (CTB–) in the pBA also contained only increased numbers of GFP+ cells in the FC group (GFP+CTB– *p* = 0.0036^q^; Fig. 3*I*). The numbers of Zif+ and GFP+Zif+ cells were not significantly different between the two groups (Zif+CTB– *p* = 0.41^r^; GFP+Zif+CTB–, HC vs chance *p* = 0.58, FC vs chance *p* = 0.52^s^; GFP+Zif+CTB– minus chance level, HC vs FC *p* = 0.41^t^; Fig. 3*J–L*). Interestingly, the dBNST-projecting neurons (CTB+) originating in the pBA were activated during contextual fear conditioning, exhibiting higher numbers of GFP+ cells in the FC group (*p* = 0.025^u^; Fig. 3*M*). There was no increase in Zif+ cells (*p* = 0.47^v^) or GFP+Zif+ cells above chance level (HC vs chance *p* = 0.84, FC vs chance *p* = 0.82^w^; GFP+Zif+ minus chance level, HC vs FC *p* = 0.48^×^) within the CTB+ population of the pBA (Fig. 3*N–P*). Our data therefore indicate that the pBA → dBNST projection, although activated during contextual fear conditioning, is not part of the contextual fear engram.

**Figure 4.**
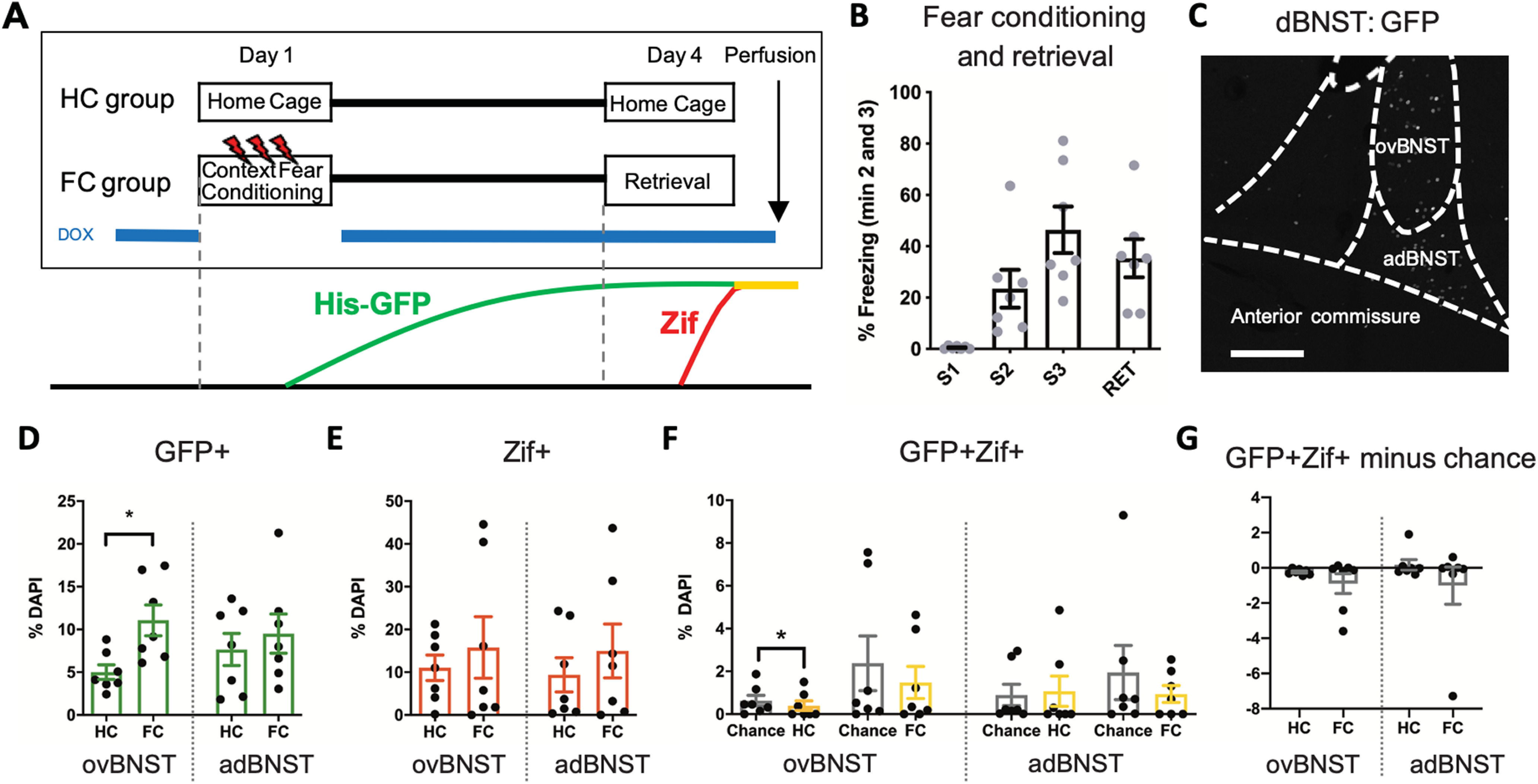
The ovBNST is active during contextual fear conditioning but is not incorporated into the fear engram. ***A***, Experimental outline for FC (*n* = 7) versus HC (*n* = 7) groups. Lower panel shows the corresponding expression timeline of the His-GFP and Zif proteins. ***B***, The FC group showed typical levels of freezing during fear conditioning trials (S1, S2, S3) and retrieval session (RET). Freezing percentages are an average of the second and third minutes of each session. ***C***, His-GFP expression in the dBNST after fear conditioning. Scale bar = 200 μm. ***D***, Fear conditioning increased the number of GFP+ fear neurons in the ovBNST (*p* = 0.011), but not in the adBNST (*p* = 0.54). ***E***, There was no significant difference in the number of Zif+ cells after retrieval in the FC group in either the ovBNST (*p* = 0.90) or adBNST (*p* = 0.71). ***F***, There was no significant difference in the number of GFP+Zif+ cells compared with chance in the ovBNST (*p* = 0.094) or adBNST (*p* = 0.56) in the FC group. ***G***, In neither region was the number of GFP+Zif+ cells minus chance level significantly different between groups (ovBNST *p* = 0.62; adBNST *p* > 0.99). Graphs show mean ± SEM. *p < 0.05.

We performed an additional experiment with separate groups of TetTag mice to investigate the activation profile of the dBNST during fear conditioning and retrieval (Fig. 4*A*). All mice in the FC group showed normal levels of freezing during fear conditioning trials and the retrieval session (Fig. 4*B*). As subdivisions of the dBNST are reported to play different roles in anxiety, we quantified the numbers of GFP+ fear neurons in the ovBNST and adBNST subnuclei separately (Fig. 4*C*). In the ovBNST, we found that the FC group (*n* = 7) had a significant increase in GFP+ neurons when compared with the HC group (*n* = 7), indicating activation of ovBNST neurons during contextual fear conditioning (*p* = 0.011^y^; Fig. 4*D*). In contrast, contextual fear conditioning had no effect on the number of GFP+ neurons in the adBNST (*p* = 0.54 ^cc^; Fig. 4*D*). Neither the ovBNST nor the adBNST showed any differences in Zif+ (ovBNST, *p* = 0.90^z^; adBNST, *p* = 0.71^dd^), or an increase in GFP+Zif+ cells above chance level (ovBNST: HC vs chance *p* = 0.016, FC vs chance *p* = 0.094^aa^, HC vs FC minus chance *p* = 0.62^bb^; adBNST: HC vs chance *p* = 0.58, FC vs chance *p* = 0.56^ee^, HC vs FC minus chance *p* > 0.99^ff^; Fig. 4*E–G*). These data reveal that, although contextual fear conditioning activates the ovBNST, neither the ovBNST nor the adBNST are significantly reactivated during expression of contextual fear. Notably, the activation patterns of the dBNST-projecting neurons in the pBA (Fig. 3*I–L*) mirrored the activation patterns of the ovBNST neurons (Fig. 4*D–G*), with both being activated during contextual fear conditioning without being reactivated during retrieval.

**Figure 5.**
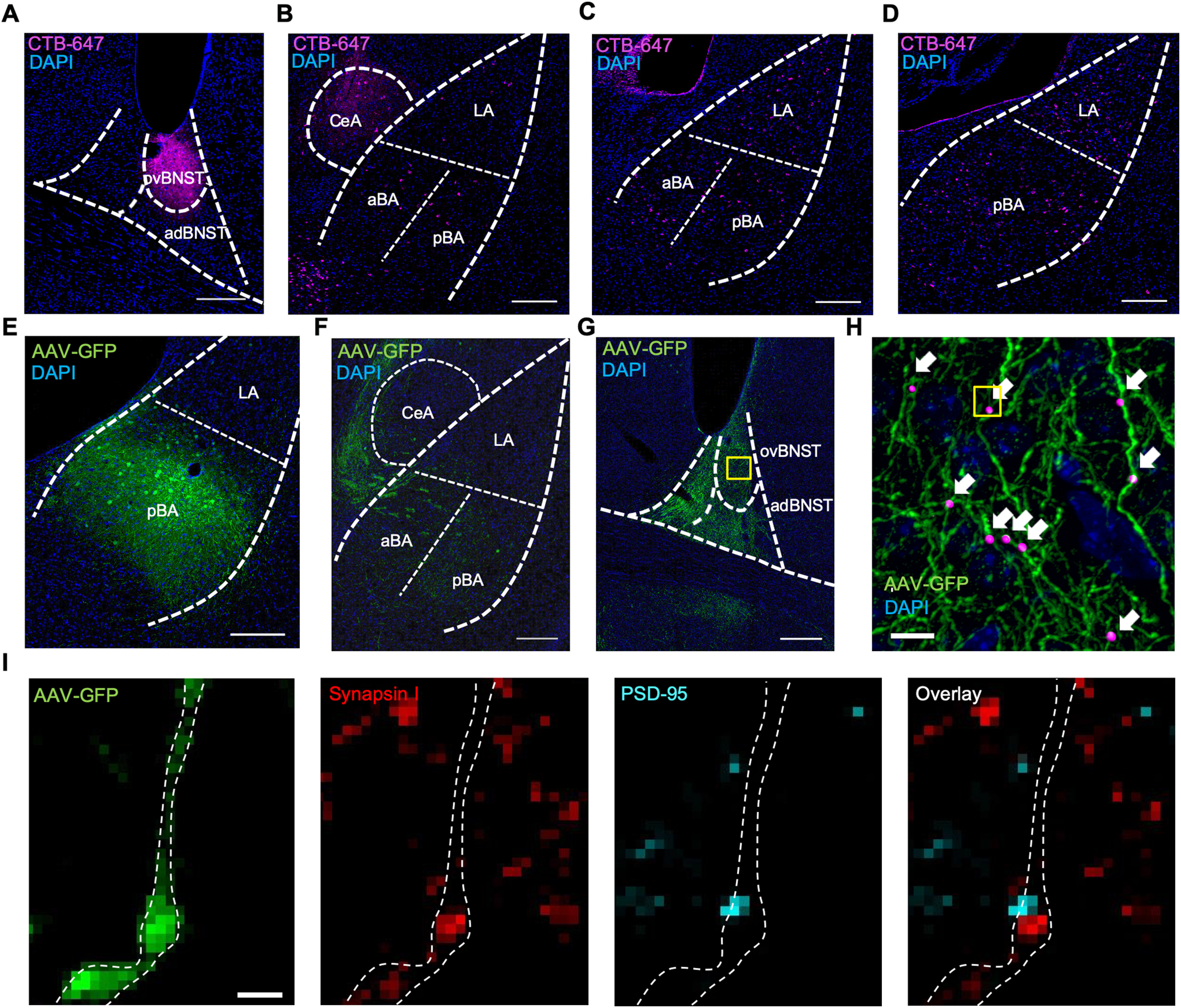
The BA projects to and makes synaptic contact with the ovBNST. ***A***, Retrograde tracer CTB-647 at the injection site in the ovBNST. ***B–D***, Retrogradely labeled CTB+ soma in the aBA, pBA, CeA, and lateral amygdala (LA). Scale bars for ***A–D*** = 200 μm. ***E***, Anterograde tracer AAV-GFP expression in the soma of BA neurons near injection site. ***F***, GFP+ axonal projections from the BA coursing around the CeA through the stria terminalis. ***G***, GFP+ axonal projections from the BA in the ovBNST and adBNST subdivisions. Scale bars for ***E–G*** = 200 μm. ***H***, 3D rendering of colocalization of SynapsinI and PSD95 at sites of GFP+ BA projections in the ovBNST (purple spheres indicated by white arrows). Yellow box in ***G*** indicates approximate image location. Scale bar = 10 μm. ***I***, Confocal image of representative SynapsinI/PSD-95 colocalization at site of GFP+ axon. Scale bar = 1 μm. Yellow box in ***H*** indicates image area.

To determine whether the similar activation patterns of pBA → dBNST neurons and ovBNST neurons could be the result of a direct projection from the BA to the ovBNST, we injected CTB into the ovBNST (Fig. 5*A*), which led to CTB-labeled soma in the BA (Fig. 5*B–D*). To further characterize this BA → ovBNST pathway, we performed an anterograde tracing experiment to determine whether BA → ovBNST projection neurons make synapses within the ovBNST. We injected an AAV2-GFP tracer virus to label axonal projections originating from cells in the BA. We imaged the BNST from a mouse in which the virus was restricted to the BA (Fig. 5*E*,*F*). We observed axonal processes in both the adBNST and the ovBNST (Fig. 5*G*). To confirm synaptic contact of the BA-originating axons in the ovBNST, we performed a colocalization study with presynaptic and postsynaptic markers, SynapsinI and PSD-95, respectively. We found multiple sites of SynapsinI/PSD-95 colocalization along GFP-labeled axon in the ovBNST, indicating that BA projection neurons send axons that make synaptic contact within the ovBNST (Fig. 5*H*,*I*).

## Discussion

In this study, we identified a population of non-dBNST-projecting neurons in the aBA that was activated during both contextual fear conditioning and contextual fear retrieval, and therefore was likely participating in the contextual fear engram. These cells were functionally and spatially distinct from a population of neurons in the pBA that was only activated during contextual fear conditioning. A portion of these activated cells in the pBA projected to the dBNST. Our data might reflect the activation of a direct monosynaptic projection from the pBA to the ovBNST during the initial encoding and/or consolidation of a contextual fear memory trace.

The amygdala has long been known to house critical elements of fear engrams (Josselyn and Tonegawa, 2020). More recently, studies have shed light on how the heterogeneous cellular population in the BA is distinct in function according to genetic makeup, location, and projection. Our current finding that fear engram neurons are preferentially located in the aBA, and not the pBA, is in line with previous studies that indicate the importance of the anterior subdivision in contextual fear memory (Goosens and Maren, 2001; Kim et al., 2016). Kim et al. (2016) identified two spatially separate populations of cells in the BA that supported either positive or negative stimuli, and their appropriate behavioral response. Specifically, they found that the aBA contained genetically distinct neurons that were incorporated into the fear engram, while the pBA did not (Kim et al., 2016). They further showed that these fear engram neurons in the BA preferentially project to the capsular nucleus of the central amygdala (CeA) and the prelimbic prefrontal cortex. It is likely that this population of cells overlaps to a high degree with the population of fear engram neurons identified in our study, which are located in the aBA and project to brain regions other than the dBNST.

While it does not appear that the BA → dBNST pathway is a direct component of the contextual fear engram, the circuit between the pBA and dBNST may function in contextual fear conditioning. Since this pathway is not reactivated during retrieval of the fear memory, it likely contributes to the encoding and/or consolidation rather than the storage of the fear memory trace. We propose that the majority of dBNST-projecting neurons in the pBA that are activated during contextual fear conditioning project to the ovBNST and not the adBNST. Our data support the hypothesis that the BA → ovBNST pathway is recruited by contextual fear conditioning for two main reasons. First, the activation pattern of the dBNST-projecting BA neurons, i.e., activation during conditioning without reactivation during retrieval, was mirrored by ovBNST neurons, but not by the adBNST neurons. Combined with the observed monosynaptic excitatory projection from BA to ovBNST (Fig. 5), which is in agreement with a recent study (Ye and Veinante, 2019), this suggests that ovBNST neurons are a direct downstream target of contextual fear conditioning-activated BA neurons. Second, the ovBNST is believed to be an anxiogenic structure while the BA → adBNST circuit is anxiolytic (Kim et al., 2013). Our finding that the ovBNST, but not the adBNST, is activated by contextual fear conditioning seems in agreement with the reported opposite functions of these subdivisions in anxiety.

It is not immediately clear why the BA → ovBNST pathway would be active during contextual fear conditioning, without being incorporated into a lasting fear memory trace as indicated by its inactivity during retrieval. A recent study found that inactivation of the BNST attenuated retrieval of a contextual fear memory if the animals received a delayed foot shock (9 min after context placement) during training, but not if the foot shock was more tightly paired with placement in the context (1 min after context placement). This suggests that the level of participation of the BNST in retrieval of contextual fear is dependent on the imminence of the perceived threat (Goode et al., 2020). In our current study, we used a 3-min delay between context placement and delivery of the first foot shock during training, which may explain why the BA-BNST circuit was not reactivated during fear retrieval. We hypothesize that the BA → ovBNST pathway plays a role in the acquisition or consolidation of the contextual fear memory when the perceived threat is imminent or predictable by exerting a modulatory effect on the fear engram. The ovBNST might exert such a modulatory effect through one or more of its downstream brain structures (Dong et al., 2001b). A monosynaptic projection from ovBNST neurons to serotonin neurons located in the dorsal raphe nucleus (DRN) has been reported (Pollak Dorocic et al., 2014; Weissbourd et al., 2014), and may be relevant given the role of serotonin in the modulation of contextual fear memories (Bauer, 2015). Serotonin from the DRN activates neurons in the BNST that evoke fear behavior through the local suppression of anxiolytic ventral BNST projections (Jennings et al., 2013; Marcinkiewcz et al., 2016), providing potential sites where the fear engram may be regulated. The adBNST also participates in local BNST microcircuits and receives monosynaptic inhibitory input from the ovBNST (Kim et al., 2013). However, despite its well-characterized anxiolytic role (Kim et al., 2013), if and how the adBNST plays a role in contextual fear is currently unclear. Finally, the ovBNST is reciprocally connected with the CeA (Dong et al., 2001a,b). The ovBNST might therefore directly modulate the formation of the contextual fear memory trace within the CeA (Goosens and Maren, 2001; Koo et al., 2004; Zimmerman et al., 2007; Pitts et al., 2009). Future studies aimed at determining where contextual fear conditioning-activated ovBNST fear neurons project to might elucidate how activation of a BA → ovBNST pathway can modulate contextual fear memory storage.

Our study has some limitations that must be considered when interpreting the results. Because of the absence of a context-only (no-shock) group for comparison, we cannot exclude certain factors that may have contributed to the activation of tagged cells other than fear conditioning. For example, the possibility remains that the GFP+ cells in the pBA were not activated during the fear conditioning sessions themselves, but on the mouse’s return to the home cage after the fear conditioning sessions. If this were the case, it is more likely that these pBA cells project to the anxiolytic adBNST rather than the anxiogenic ovBNST and may belong to a predefined population of cells that play a role in reward behavior (Zhang et al., 2020). Alternatively, the GFP-tagged cells in the pBA could have been activated due to the novel experience and handling, although this is less likely since strong activation of BA cells through robust fear conditioning was found to be required for significant tagging in the TetTag mouse (Reijmers et al., 2007). Additional experiments are therefore needed to clarify the function of the dBNST-projecting cells in the pBA, and their reason for activation. Another caveat to this study is the inability to distinguish between cells active in the HC group and the FC group, which could be comprised of different subpopulations of cells with distinct functions. Additionally, it is possible that not all BNST-projecting neurons were labeled by the CTB injection, underestimating the active cells in this pathway.

In summary, our study confirms functionally distinct and projection-specific cellular populations in the BA. Specifically, we observed that the role of BA projection neurons in contextual fear memory differentiates across BA subdivisions (aBA vs pBA) and across downstream projection targets (dBNST vs non-dBNST). Fear engram cells were preferentially localized in the aBA and did not project to the dBNST. Furthermore, our data point to a pBA → ovBNST pathway that is activated during contextual fear conditioning, but that is not incorporated into the fear engram. Future optogenetic and pharmacogenetic studies of the BA → ovBNST pathway can directly test its necessity and sufficiency for the initial storage of contextual fear memories.

## Synthesis

Reviewing Editor: Thomas McHugh, RIKEN Center for Brain Science Decisions are customarily a result of the Reviewing Editor and the peer reviewers coming together and discussing their recommendations until a consensus is reached. When revisions are invited, a fact-based synthesis statement explaining their decision and outlining what is needed to prepare a revision will be listed below. The following reviewer(s) agreed to reveal their identity: Kurt Fraser.

This manuscript details the activation of basolateral amygdala neurons during contextual fear conditioning and retrieval. The authors used the TetTag transgenic mouse to label neurons active during conditioning, Zif268 as a marker to visualize cells activated during retrieval and a retrograde tracer, CTB-Alexa647 to label BLA neurons based on projection targets. They identify anterior-posterior differences in the patterns of basolateral amygdala activation, with anterior BLA neurons not projecting to the dorsal BNST active during conditioning and retrieval and posterior BLA neurons projecting to the BNST active only during conditioning. Based on the results, this study concludes that dBNST-projecting neurons in the BLA do not appear to participate in contextual fear engrams and a BLA-ovBNST pathway may be involved in encoding process of contextual fear memory. The authors also provide evidence for a potential synaptic connection between the posterior BLA and the dBNST.

Upon discussion the reviewers agreed that the manuscript was interesting, however they raised several points that should be addressed before further consideration. These points are explained below.

1. Clarifications of conclusions, addressing of limitations and improvements in the text

a. The terminology of engram neuron and fear neuron needs to be clearly defined and used in the text.

b. The absence of a context-exposed group in these experiments prevents a total exclusion of the many factors other than footshock/conditioning contributing to neuronal activation. This issue should be addressed in the discussion.

c. Related to the point above, the imaging analysis used in this study cannot distinguish cells active during learning or retrieval from those active in the homecage. This limitation makes it difficult to interpret some of the data. In Figure 2G, for example, there are some BNST-projecting cells in the aBA double positive for GFP and Zif signal. According to the concept of methods described in the first paragraph of results section, those double positive cells should represent the cells active during learning that were reactivated during retrieval session. Although there is no significant difference in percentage between HC and FC group, it is possible that different subpopulations of cells were active in each condition. Thus, the claim that contextual fear engram neurons in the aBA do not project to the dBNST can be misleading. Similar concern about the interpretation of data presented in Figure 3; it seems relatively clear that there is a subset of non-BNST projecting cells in the aBA that was activated during FC and reactivated during later retrieval. This caveats/limitations should be addressed in the discussion.

d. Related with the above issue, the interpretation of data is further limited due to the possibility that not all BNST-projecting BA neurons are labeled by CTB injection.

2. Clarification of methods and experimental design

a. I could not find details on the handling of homecage mice, were they brought to the conditioning room, were they exposed to the context at retrieval, what was their perfusion timeline, etc.; please add to the methods.

b. I am curious about the inclusion of only 1 animal for the final experiments analyzing synaptic connectivity. What was the exclusion criteria for other cases? Why was just one animal used? If this is standard subject size then references justifying this are needed.

c. For contextual fear conditioning, the authors used 3 training sessions each separated by 3 hours. Please explain the rationale for this protocol.

3. Improvements in figures and data

a. It would improve the manuscript to also see presented the total GFP and ZIF active neurons in each BLA region absent the CTB criterion. This first analysis would be nice to have and could prove compelling to readers more generally interested in these anterior-posterior differences as this is an emerging area of interest in the field. As it is, it is not obvious how to interpret the justification for exclusion of CTB being the default for total anterior or posterior activation.

b. In Figure 2 and 3, representative images should be provided.
